# Fas receptor is expressed in human lung squamous cell carcinomas, whereas bcl-2 and apoptosis are not pronounced: a preliminary report.

**DOI:** 10.1038/bjc.1997.359

**Published:** 1997

**Authors:** H. B. Hellquist, B. Olejnicka, M. Jadner, T. Andersson, C. Sederholm

**Affiliations:** Department of Pathology II, University Hospital, LinkÃ¶ping, Sweden.

## Abstract

**Images:**


British Journal of Cancer (1997) 76(2), 175–179
© 1997 Cancer Research Campaign

### Fas receptor is expressed in human lung squamous cell carcinomas, whereas bcl-2 and apoptosis are not pronounced: a preliminary report

**HB Hellquist[1], B Olejnicka[1], M Jadner[2], T Andersson[1] and C Sederholm[3]**

[1]Department of Pathology II, [2]Department of Otolaryngology and [3]Department of Lung Medicine, University Hospital, Linköping, Sweden

**Summary** We report a pilot study on the Fas receptor (APO-1, CD95) in vivo in 15 human squamous cell (non-small) carcinomas and ten normal bronchial specimens. The principal aim was to investigate whether the so-called death receptor, Fas, is present in these tumours. Ligation of Fas promptly induces apoptosis, particularly in T Jurkat cells in vitro, and expression of Fas on human cancer would thus theoretically be of great interest. The immunoreactivity for the anti-apoptotic protein Bcl-2 was also investigated, and the degree of apoptosis was evaluated by TdT dUTP nick end labelling (TUNEL) and conventional morphological criteria. Fas was present in all initial tumours but absent in control tissue, that is in the potential precursor cells of bronchial epithelium ($P = 0.001$). Fas was not detectable after radiotherapy ($P = 0.03$). We propose that radiotherapy induces an early selection of tumour cells rather than a down-regulation of Fas. Both Bcl-2 and apoptosis (TUNEL) were generally expressed at a modest level. In agreement with other studies, we did not find any significant correlation between Bcl-2 and prognosis, or between Bcl-2 and TUNEL. Hence, in this preliminary report, we have demonstrated Fas receptor in human squamous cell carcinomas in vivo. This is a novel finding, and the apparent absence of Fas after radiotherapy may have important therapeutic implications.

**Keywords:** Fas; CD95; apoptosis; bcl-2; lung cancer; in situ hybridization; immunocytochemistry

Squamous cell carcinoma accounts for approximately 35% of all human lung carcinomas and originates often in a central or hilar location (Faber, 1991). The principal non-surgical therapeutic aim is either to reduce tumour cell proliferation rate and/or to increase tumour cell death, i.e. to reduce the net tumour growth. The two main mechanisms of cell death are necrosis and apoptosis (Wyllie, 1985). Apoptosis has a major influence on net tumour growth (Arends et al, 1994) and is characterized by successive fragmentation of DNA, which in most cell types eventually results in 180-bp-sized fragments. Ligation with the Fas ligand to the Fas receptor (APO-1, CD95) induces apoptosis, particularly in cultured human T Jurkat cells but also in human B cells, cultured human rhabdomyosarcoma cells (Yonehara et al, 1989) and murine fibroblast L929 cells and T-cell lymphoma WR19L cells (Itoh and Nagata, 1993; Firestein et al, 1995). So far, the Fas receptor has not been investigated in depth in human cells in vivo. In the literature, we have found only a few papers reporting Fas in vivo, e.g. on certain fibroblasts and fibroblast-like synoviocytes and on neuroblastoma tumour cells (Aggarwal et al, 1995; Firestein et al, 1995; Koizumi et al, 1995). Its possible presence on human cell types other than T cells may open new approaches concerning both research and eventually therapy.

In this study, we have also included immunocytochemical investigation of the Bcl-2 protein. The *bcl*-2 survival proto-oncogene encodes for a 26-kDa protein that protects cells from apoptosis, favouring a prolonged survival of normal as well as neoplastic cells (Vaux et al, 1988; Nunez et al, 1990; Pezzella et al, 1993; Doglioni et al, 1994; Pilotti et al, 1994). This protein, in a way similar to metalloproteins, can hinder the apoptotic transduction signals. As a measure of apoptosis, we applied TUNEL (TdT dUTP nick end labelling) to the carcinomas and related the findings with morphological signs of apoptotic death.

We report that all carcinomas expressed the Fas receptor, while most specimens from non-neoplastic control bronchial tissue lacked Fas, and Fas apparently disappears during radiotherapy. The Bcl-2 protein varied between tumours but was relatively sparsely expressed. Apoptosis, evaluated by TUNEL, was only moderately pronounced in some tumours, before treatment as well as after treatment.

*Received 22 July 1996*
*Revised 29 October 1996*
*Accepted 22 January 1997*

*Correspondence to:* HB Hellquist, Department of Pathology II, Faculty of Health Sciences, Linköping University, S-581 85 Linköping, Sweden

### MATERIALS AND METHODS

### Tissue samples

Biopsy specimens were obtained by bronchoscopy from eight patients with bronchial squamous cell carcinoma. We felt it imperative to obtain initial biopsies before any kind of treatment and also to be able to perform a biopsy after the therapy given. Hence, this study included a rather small number of patients, however statistical analysis was still possible. In three of the patients with carcinoma, macroscopical normal bronchial mucosal biopsies from the contralateral non-neoplastic lung were obtained. Specimens were thus taken both before and after therapy (fractionated radiotherapy in cases 2, 3, 4, 6 and 7 and low-dose-rate brachytherapy in cases 1 and 5). One patient has not yet received radiotherapy. Bronchial muscosal biopsy specimens were also obtained from seven subjects with non-neoplastic, non-inflammatory lung disease.

**Figure 1** (**A**) Positive immunoreactivity for Fas in a moderately well-differentiated lung squamous cell carcinoma (case 5, Fas, Clone UB-2, Oncor, USA, × 450). (**B**) A squamous carcinoma with a moderate Bcl-2 staining intensity, although the extent of the immunoreactivity is high (case 2, Bcl-2, DAKO, Denmark, × 450). (**C**) Photomicrograph of a squamous cell carcinoma in which several cells show nuclear TUNEL positivity. Note the apoptotic morphology of TUNEL-positive cells (case 6, TUNEL, Oncor, USA, × 380)

Because of the small size of most biopsy specimens, we were only able to cut a few sections in most cases. During processing (microwave heating, etc.) some sections were lost, and the ability to repeat certain investigations was not always possible.

### Immunocytochemistry and TUNEL

For each case, 4 to 5-µm-thick sections were cut from representative blocks. One section was routinely stained with haematoxylin and eosin, and other sections were placed on pretreated slides (poly-L-lysine) for later heating in a microwave oven before immunostaining and TUNEL. All slides were coded and the examiners had no access to clinical data.

Sections for immunocytochemistry were treated with 3% hydrogen peroxide in distilled water to inhibit endogenous peroxidase activity and then immersed in boiling citrate buffer, pH 6, in a microwave oven (Shi et al, 1991) with two changes of 7 min each. After washing in tap water and distilled water, the sections were subsequently incubated with (1) 1:20 dilution of normal rabbit serum for 20 min at room temperature, (2) 1:25 dilution of the monoclonal anti-Fas antibody (anti-human Fas, Clone UB-2, murine, Oncor, Gaithersburg, MD, USA) and 1:50 dilution of the monoclonal antibody to Bcl-2 protein, overnight at +4°C, (3) 1:200 dilution of a biotinylated rabbit antiserum to mouse immunoglobulins for 30 min at room temperature and (4) 1:100 dilution of the streptavidin-biotinylated peroxidase complex for 30 min, also at room temperature. Peroxidase activity was developed in diaminobenzidine (DAB) chromogene substrate. All reagents were bought from Dako (Copenhagen, Denmark).

A known positive control for Bcl-2 [a formalin-fixed, paraffin-embedded follicular lymphoma carrying the (t 14;18) chromosomal translocation] was immunostained with the test samples. Neoplastic cells showing definite cytoplasmic staining of the same intensity, or higher, as shown by the lymphoma cells, were graded as 3+. Tumour cells with moderate intensity staining reaction were graded 2+, weakly positive cells 1+ and tumour cells lacking immunoreactivity were recorded as negative. In most tumours, there were scattered lymphocytes, which served as further internal positive controls. Negative control sections (in which the specific monoclonal antibody was substituted with the immunoglobulin fraction of non-immune mouse sera) remained unstained. The extent of immunoreactivity was evaluated as 1+ if less then 25% of tumour cells were positive, 2+ if 25–75% were positive and 3+ if more than 75% of cells were positive. Only tumours with cells showing an intensity of 2+ and 3+ were recorded as positive.

T Jurkat cells were used as positive controls for Fas immunostaining (Jurkat E6.1, ECACC number 88042803, CAMR, Salisbury, Wiltshire, UK). T Jurkat cells were cultured, spun to a pellet, fixed in 4% formalin and embedded in paraffin. Sections were cut, treated identically to the test samples and used in each run as positive controls for Fas. Another monoclonal antibody was also tested and used in every sample (clone CH-11, Oncor, Gaithersburg, MD, USA); furthermore, both antibodies were tested repeatedly on frozen lung squamous carcinoma, all with a satisfactory result. Fas was registered as positive in test samples if the immunoreactivity had the same, or almost equal, intensity to that seen in the Jurkat pellet sections.

Sections for the TUNEL reaction were after deparaffinization digested by 20 µg $ml^{-1}$ proteinase K for 15 min. After four washes in distilled water and quenching in 2.0% hydrogen paroxide, the

 © Cancer Research Campaign 1997

**Table 1** Immunoreactivity of Fas and Bcl-2, and end labelling (TUNEL) in human lung carcinomas and healthy bronchial mucosae

| Case | Fas | Bcl-2 | TUNEL | Comment (biopsy before and after therapy) |
|---|---|---|---|---|
| 1 | Pos | ++ | +++ | Initial biopsy |
| | Neg | + | Neg | After radiotherapy |
| 2 | Pos | ++ | +++ | Initial biopsy |
| | Neg | ++ | +++ | After radiotherapy |
| 3 | Pos | +++ | + | Initial biopsy |
| | Pos | +++ | ++ | After radiotherapy |
| 4 | Pos | Neg | Lost | Initial biopsy |
| | Neg | Neg | ++ | After radiotherapy |
| 5 | Pos | ++ | Neg | Initial biopsy |
| | Neg | Neg | +++ | After radiotherapy |
| 6 | Pos | Neg | +++ | Initial biopsy |
| | Pos | Neg | Neg | After radiotherapy |
| 7 | Pos | ++ | Neg | Initial biopsy |
| | Neg | Neg | +++ | After radiotherapy |
| 8 | Pos | + | +++ | Initial biopsy |
| *Controls* | | | | |
| A | Neg | Lost | Neg | |
| B | Pos | + | Neg | |
| C | Neg | Neg | Neg | |
| D | Neg | Neg | Neg | |
| E | Neg | Neg | Neg | |
| F | Neg | +++ | Neg | |
| G | Pos | ++ | Neg | |
| H | Neg | Neg | +++ | Contralateral bronchus to case 5 |
| I | Neg | Neg | Lost | Contralateral bronchus to case 6 |
| J | Neg | Neg | Neg | Contralateral bronchus to case 7 |

Pos, positive; neg, negative; lost, sections lost during staining procedures. Bcl-2: +, < 25% of cells positive; ++, 25–75% positive; and +++, > 75% positive. TUNEL: +, < 5% of cells positive; ++, 5–25% positive; and +++, > 25% positive cells.

Apoptag kit was applied according to the manufacturer's instructions (Oncor, Gaithersburg, MD, USA). Briefly, the TUNEL method is a tailing reaction in which residues of digoxigenin nucleotide are analytically added to DNA by terminal deoxynucleotidyl transferase (TdT). The incorporated nucleotides form a random heteropolymer of digoxigenin-11-dUTP and dATP in a ratio that has been optimized for anti-digoxigenin antibody binding. The positivity was evaluated as 1+ when < 5% of tumour cells showed a distinct nuclear staining (with an intensity equal or almost equal to that of the control), 2+ if > 5% but less than 25% were positive and 3+ when more than 25% of tumour cells were positive. Only TUNEL-positive cells with chromosome condensation and nuclear fragmentation were regarded as positive, thus confirming the good and appropriate use of this technique. Tumours with no positive cells, or the occasional positive cell only, were recorded as negative. Samples in which TdT was substituted with water served as negative controls and, as positive control, DNAase I was added to the samples (20 min at 37°C). The DNAase was added after quenching in hydrogen peroxide, thus producing DNA breaks in virtually all cells (Gavrieli et al, 1992).

### Statistics

Fischer's exact test was used for analysis of differences in expression of Fas (and TUNEL) in initial cancer specimens and controls, and the McNemar test was used for analysis of differences between initial cancer specimens and specimens from treated tumours. The Wilcoxon test was used for analysis of differences between initial specimens and specimens from treated tumours concerning TUNEL. The Spearman rank correlation test was used for investigation of any correlation between Bcl-2 and TUNEL.

### RESULTS

### Fas

The Fas receptor was present in all eight initial squamous cell carcinoma biopsies, with an intensity equal or almost equal to that of the T Jurkat pellet control sections (Figure 1A). Fas was only found in two of the ten non-neoplastic bronchial mucosal biopsies (Table 1) ($P < 0.001$). Fas was absent in five of the seven biopsies taken from residual (or recurrent) tumour after treatment. (In one case, no biopsy has been taken after treatment, i.e. case 8.) Fas was not detectable in tumours after radiotherapy ($P = 0.03$). None of the tumours show morphological evidence of necrosis. The ten control biopsies showed a mixture of respiratory epithelium and metaplastic squamous epithelium, and all lacked morphological atypia.

### Bcl-2

There was no significant difference in the expression of Bcl-2 between the initial carcinoma specimens and the controls ($P = 0.12$). In five initial tumours, more than 25% of the cells were Bcl-2 positive (Figure 1B), and two of the controls were positive. Two of these five cases remained Bcl-2 positive after radiotherapy and three became negative. In none of the cases did Bcl-2 become upregulated after treatment.

### Tunel

TUNEL was strongly expressed in four of the eight initial carcinoma biopsies (Figure 1C), was expressed in one and negative in two (one specimen was lost during processing). It was present in one of the controls ($P = 0.08$). After treatment, only three cases showed TUNEL; of which two did not show TUNEL before treatment (cases 5 and 7). Two cases (cases 1 and 6) showed the reverse situation, i.e. they apparently lost their TUNEL positivity after given therapy (Table 1). Importantly, all cases with TUNEL positivity showed conventional morphological criteria of apoptosis. There was no difference in the expression of TUNEL between initial tumour specimens and specimens taken after treatment ($P < 0.89$). There was no positive correlation between expression of Bcl-2 and TUNEL ($P = 0.5$). All tumours were moderately to well-differentiated squamous cell carcinomas, and TUNEL paralleled conventional morphological criteria for apoptosis.

### DISCUSSION

Several anti-cancer drugs and radiotherapy act via a cascade of biochemical events of which several eventually induce apoptosis (Ijiri and Potten, 1983; Meyn et al, 1993; Ling et al, 1994; Dewey et al, 1995; Moreira et al, 1995; Stapper et al, 1995). Ligation of Fas by anti-Fas antibodies or its specific ligand rapidly induces

© Cancer Research Campaign 1997 

apoptosis in vitro and in vivo (Trauth et al, 1989; Itoh et al, 1991; Ogasawara et al, 1993), and some cytotoxic T cells kill their targets in a calcium-independent manner via activation of Fas (Rouvier et al, 1993). Most of the signal transduction events distal to Fas ligation have yet not been fully elucidated but several reports indicate that Fas-induced apoptosis is mediated via a ceramide-initiated *ras* signalling pathway (Gulbins et al, 1995). In the present study, we did not study transduction signals but investigated Fas, Bcl-2 and apoptosis in human squamous cell carcinoma cells in vivo. The results indicate that Fas is present in lung squamous cell carcinomas but not in the potential precursor cell ($P < 0.001$). This has not been reported before. In the specimens taken after radiotherapy, Fas was apparently lacking ($P = 0.03$). We interpret this as a selection of tumour cells rather than a down-regulation of Fas. Certain Fas-positive tumour cells will die because of radiotherapy, and if one of the pathways to death is via the Fas receptor the absence of Fas after therapy could probably be explained by these Fas-positive cells having been selected out. If Fas is present on tumour cells in vivo and not on normal bronchial epithelial cells, this may have important therapeutic implications.

In a previous study *bcl*-1 was reported to be amplified in poorly differentiated squamous cell carcinoma of the lung (Berenson et al, 1990), but *bcl*-1 [on chromosome 11, t(11;14)(q13;q32)] is different in several respects in comparison to *bcl*-2 [on chromosome 14, t(14;18)(q32;q21)], and hence comparison is not relevant. A few reports have been published demonstrating a weak to moderate expression of Bcl-2 protein in squamous cell carcinomas of, for example, uterine cervical squamous cell carcinoma (Uehara et al, 1995) and skin carcinomas (Cerroni et al, 1994; Sleater et al, 1994). In a large study of 126 lung carcinomas, 40 cases were squamous cell carcinomas and only 25% of these expressed Bcl-2 (Ritter et al, 1995). In none of these studies was Bcl-2 shown to be an independent prognostic factor. Similarly, our present study did not show any overexpression of Bcl-2 compared with controls and nor was it shown to be of any prognostic value. Pezzella et al (1993) detected Bcl-2 in 25% of their lung squamous cell carcinomas and, to our knowledge, this is the only study in which a significant correlation has been found between the expression of Bcl-2 and prognosis. Törmänen et al (1995) reported 28% of their lung squamous cell carcinomas to be Bcl-2 positive but did not find Bcl-2 to be an independent prognostic factor. We similarly found that apoptosis (TUNEL) is not correlated with the expression of Bcl-2 ($P = 0.5$). The TUNEL positivity in our present study correlated well in all cases with apoptotic morphology. Several tumours had more than 25% TUNEL-positive cells (Table 1). This is a high apoptotic rate indeed but can probably be explained in part by the nature of the biopsy specimens. Bronchial specimens are naturally very small and may represent superficial, partly dead and almost exfoliated parts of the tumours, and they therefore show a high apoptotic rate. There are studies that indicate that overexpression of Bcl-2 protein favours a prolonged survival of normal as well as of neoplastic cells (Vaux et al, 1988; Nunez et al, 1990; Doglioni et al, 1994; Pilotti et al, 1994). Yet other studies have demonstrated that overexpression of Bcl-2 results in resistance to chemotherapy, suggesting that Bcl-2 interferes and prevents chemotherapy-induced apoptosis (Dole et al, 1994; Hashimoto et al, 1995). The present findings, i.e. the relatively low levels of Bcl-2 and the finding that Bcl-2 is not an independent prognostic factor, are thus in agreement with most previous observations. All tumours but one in the present series were treated with radiotherapy, and chemotherapy has not yet been planned or performed. Radiotherapy did apparently not affect the expression of Bcl-2 (Table 1).

The important paper by Törmänen et al (1995) shows that enhanced apoptosis predicts shortened survival in non-small-cell lung carcinoma. This finding could not be confirmed in the present series, which may have numerous explanations. They correlated apoptosis with rate of cell proliferation, immunoreactivity of p53 and Bcl-2, morphological tumour necrosis and survival data (Törmänen et al, 1995). Our principal aim was to investigate Fas and, to a lesser extent, Bcl-2 and apoptosis (by TUNEL and histology) rather than a careful statistical analysis between examined parameters and survival rate. Although a pilot study, our results indicate a novel finding that human squamous (non-small) cell carcinomas express Fas in vivo and also that the receptor is no longer detectable after radiotherapy. These observations need to be confirmed in a larger series of tumours, and also in vitro, as they may have both prognostic and theurapeutic implications.

### ACKNOWLEDGEMENTS

The financial support from Linköping University Hospital Funds, Östergötlands Läns Landsting and FORSS is gratefully acknowledged.

### REFERENCES

Aggarwal BB, Singh S, Lapushin R and Totpal K (1995) Fas antigen signals proliferation of normal human diploid fibroblast and its mechanism is different from tumor necrosis factor receptor. *FEBS* **363**: 5–8

Arends MJ, McGregor AH and Wyllie AH (1994) Apoptosis is inversely related to necrosis and determines net growth in tumors bearing constitutively expressed *myc*, *ras* and HPV oncogenes. *Am J Pathol* **144**: 1045–1057

Berenson JR, Koga H, Yang J, Pearl J, Holmes C, Figlin R and the Lung Cancer Study Group (1990) Frequent amplification of the *bcl*-1 locus in poorly differentiated squamous cell carcinoma. *Oncogene* **5**: 1343–1348

Cerroni L and Kerl H (1994) Aberrant bcl-2 protein expression provides a possible mechanism of neoplastic cell growth in cutaneous basal-cell carcinoma. *J Cutan Pathol* **21**: 398–403

Dewey WC, Ling CC and Meyn RE (1995) Radiation-induced apoptosis: relevance to radiotherapy. *Int J Radiat Oncol Biol Phys* **33**: 781–796

Doglioni C, Dei Tos AP, Laurino L, Chiarelli C, Barbareschi M and Viale G (1994) The prevalence of BCL-2 immunoreactivity in breast carcinomas and its clinicopathological correlates, with particular reference to oestrogen receptor status. *Virchows Arch* **424**: 47–51

Dole M, Nunez G, Merchant AK, Maybaum J, Rode CK, Bloch CA and Castle VP (1994) Bcl-2 inhibits chemotherapy-induced apoptosis in neuroblastoma. *Cancer Res* **54**: 3253–3259

Faber LP (1991) Lung cancer. In *American Cancer Society Textbook of Clinical Oncology*, Holleb AI, Fink DJ and Murphy GP. (eds), pp. 194–212, The American Cancer Society: Atlanta

Firestein GS, Yeo M and Zvaifler NJ (1995) Apoptosis in rheumatoid arthritis synovium. *J Clin Invest* **96**: 1631–1638

Gavrieli Y, Sherman Y and Ben-Sasson SA (1992) Identification of programmed cell death in situ via specific labeling of nuclear fragmentation. *J Cell Biol* **119**: 493–501

Gulbins E, Bissonnette R, Mahboubi A, Martin S, Nishioka W, Brunner T, Baier G, Baier-Bitterlich G, Byrd C, Lang F, Kolesnick R, Altman A and Green D (1995) FAS-induced apoptosis is mediated via a ceramide-initiated RAS signaling pathway. *Immunity* **2**: 1–20

Hashimoto H, Chatterjee S and Berger NA (1995) Inhibition of Etopside (VP-16)-induced DNA recombination and mutant frequency by Bcl-2 protein overexpression. *Cancer Res* **55**: 4029–4035

Ijiri K and Potten CS (1983) Response of intestinal cells of differing topographical and hierarchical status to ten cytotoxic drugs and five sources of radiation. *Br J Cancer* **47**: 175–185

Itoh N and Nagata S (1993) A novel protein domain required for apoptosis. *J Biol Chem* **268**: 10932–10937

© Cancer Research Campaign 1997

Itoh N, Yonehara S, Ishii A, Yonehara M, Mizushima S-J, Sameshima M, Hase A, Seto Y and Nagata S (1991) The polypeptide encoded by the cDNA for human cell surface antigen Fas can mediate apoptosis. *Cell* **66**: 233–243

Koizumi H, Wakisaka M, Nakada K, Takakuwa T, Fujioka T, Yamate N and Uchikoshi T (1995) Demonstration of apoptosis in neuroblastoma and its relationship to tumour regression. *Virchows Arch* **427**: 167–173

Ling CC, Chen CH and Fuks Z (1994) An equation for the dose responce of radiation-induced apoptosis: possible incorporation with the LQ model. *Radiother Oncol* **33**: 17–22

Meyn RE, Stephens LC, Ang KK, Hunter NR, Brock WA, Milas L and Peters LJ (1993) Heterogeneity in the development of apoptosis in irradiated murine tumours of different histologies. *Int J Radiat Biol* **64**: 583–591

Moreira LF, Naomoto Y, Hamada M, Kamikawa Y and Orito K (1995) Assessment of apoptosis in oesophageal carcinoma preoperatively treated by chemotherapy and radiotherapy. *Anticancer Res* **15**: 639–644

Nunez G, London L, Hockenbery D, Alexander M, McKearn JP and Korsmeyer SJ (1990) Deregulated Bcl-2 gene expression selectively prolongs survival of growth factor-deprived hemopoietic cell lines. *J Immunol* **144**: 3602–3610

Ogasawara J, Watanabe-Fukunaga R, Adachi M, Matsuzawa A, Kasugai T, Kitamura Y, Itoh N, Suda T and Nagata S (1993) Lethal effect of the anti-Fas antibody in mice. *Nature* **364**: 806–809

Pezzella F, Turley H, Kuzu I, Tungekar MF, Dunnill MS, Pierce CB, Harris A, Gatter KC and Mason DY (1993) *bcl*-2 protein in non-small-cell lung carcinoma. *N Engl J Med* **329**: 690–694

Pilotti S, Collini S, Rilke F, Cattoretti G, Del Bo R and Pierotti MA (1994) BCL-2 protein expression in carcinomas originating from the follicular epithelium of the thyroid gland. *J Pathol* **172**: 337–342

Ritter JH, Dresler JH and Wick MR (1995) Expression of bcl-2 in stage T1N0M0 non-small cell lung carcinoma. *Hum Pathol* **26**: 1227–1232

Rouvier E, Louvier E, Luciani MF and Goldstein P (1993) Fas involvement in Ca(2+)-independent T cell-mediated cytotoxicity. *J Exp Med* **177**: 195–200

Shi S-R, Key ME and Kalra KL (1991) Antigen retrieval in formalin-fixed, paraffin-embedded tissues: an enhancement method for immunohistochemical staining based on microwave oven heating of tissue sections. *J Histochem Cytochem* **39**: 741–748

Sleater JP, Beers BB, Stephens CA and Hendricks JB (1994) Keratoacanthoma: a deficient squamous cell carcinoma? Study of bcl-2 expression. *J Cutan Pathol* **21**: 514–519

Stapper NJ, Stuschke M, Sak A and Stube G (1995) Radiation-induced apoptosis in human sarcoma and glioma cell lines. *Int J Cancer* **62**: 58–62

Törmänen U, Eerola A-K, Rainio P, Vähäkangas K, Soini Y, Sormunen R, Bloigu R, Lehto V-P and Pääkkö P (1995) Enhanced apoptosis predicts shortened survival in non-small cell lung carcinoma. *Cancer Res* **55**: 5595–5602

Trauth BC, Klas C, Peters AMJ, Matzku S, Möller P, Falk W, Debatin K-M and Krammer PH (1989) Monoclonal antibody-mediated tumor regression by induction of apoptosis. *Science* **245**: 301–305

Uehara T, Kuwashima Y, Izumo T, Kishi K, Shiromizu K and Matsuzawa M (1995) Expression of the proto-oncogene bcl-2 in uterine cervical squamous cell carcinoma: its relationship to clinical outcome. *Eur J Gynaecol Oncol* **16**: 453–460

Vaux DL, Cory S and Adams JM (1988) *Bcl*-2 gene promotes haematopoietic cell survival and cooperates with c-*myc* to immortalize pre-B cells. *Nature* **335**: 440–445

Wyllie AH (1985) The biology of cell death in tumors. *Anticancer Res* **5**: 131–136

Yonehara S, Ishii A and Yonehara A (1989) A cell-killing monoclonal antibody (anti-fas) to a cell surface antigen co-downregulated with the receptor of tumor necrosis factor. *J Exp Med* **169**: 1747–1756

© Cancer Research Campaign 1997


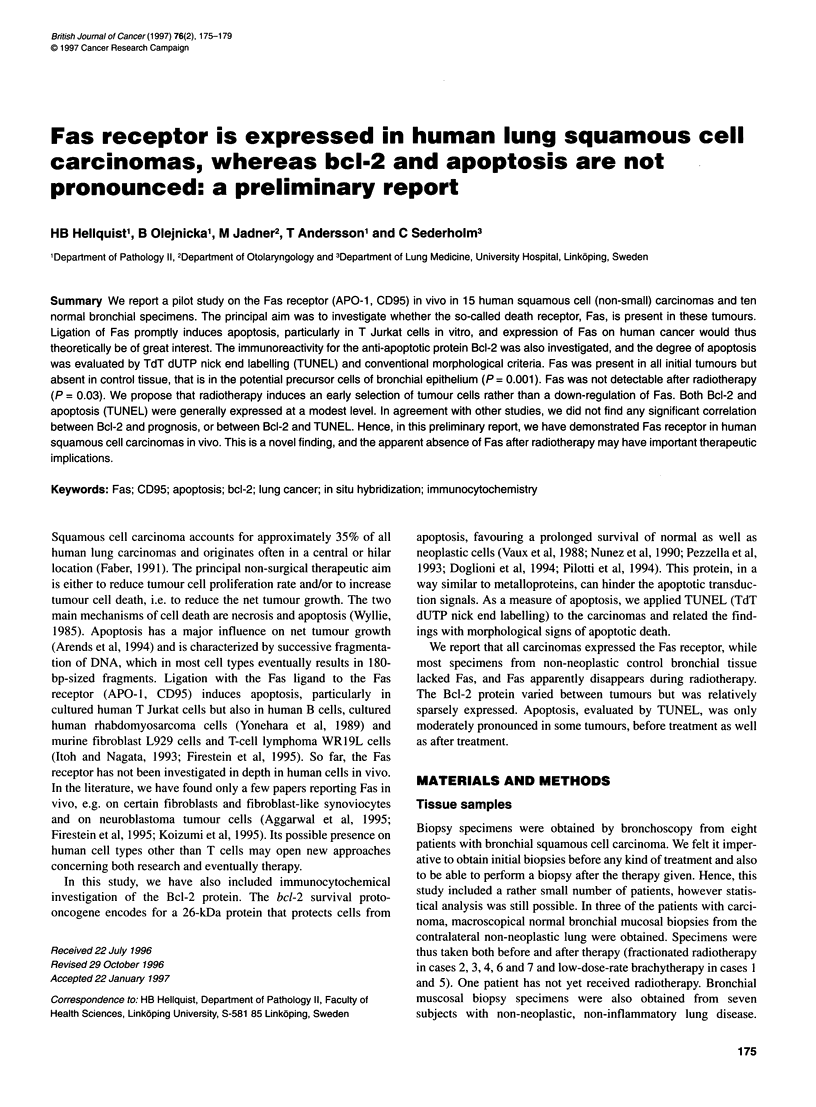

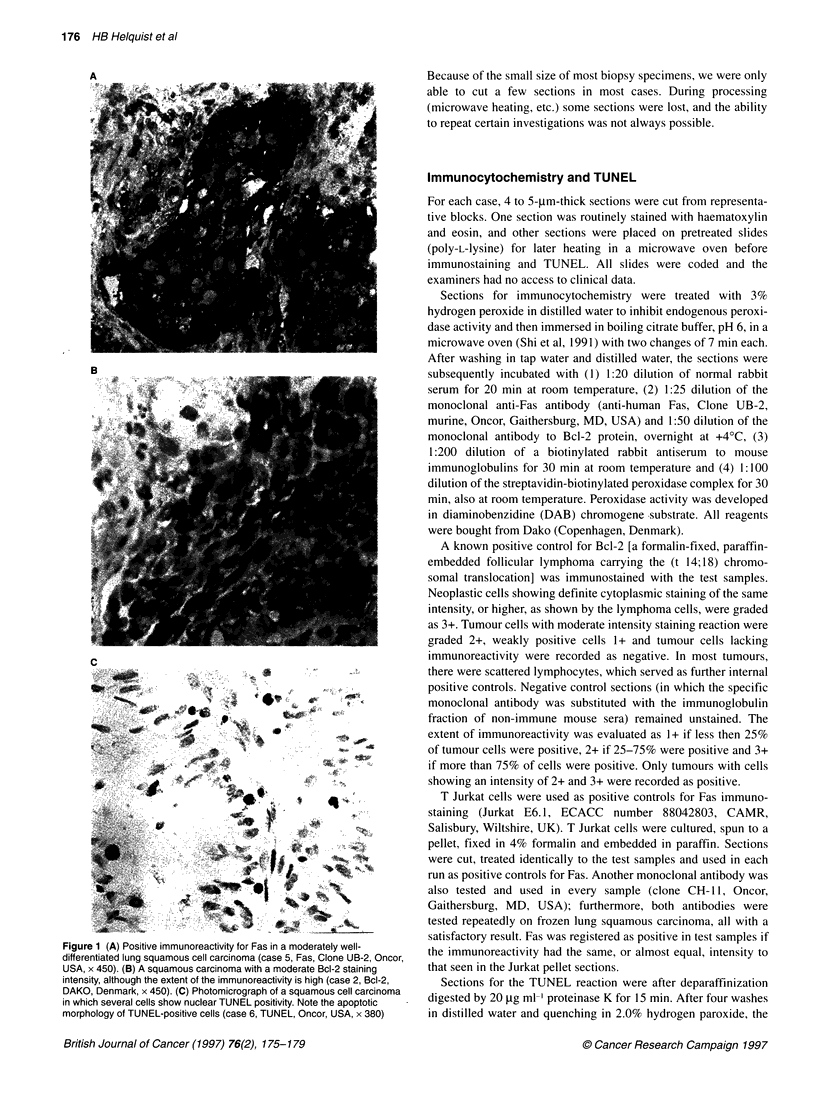

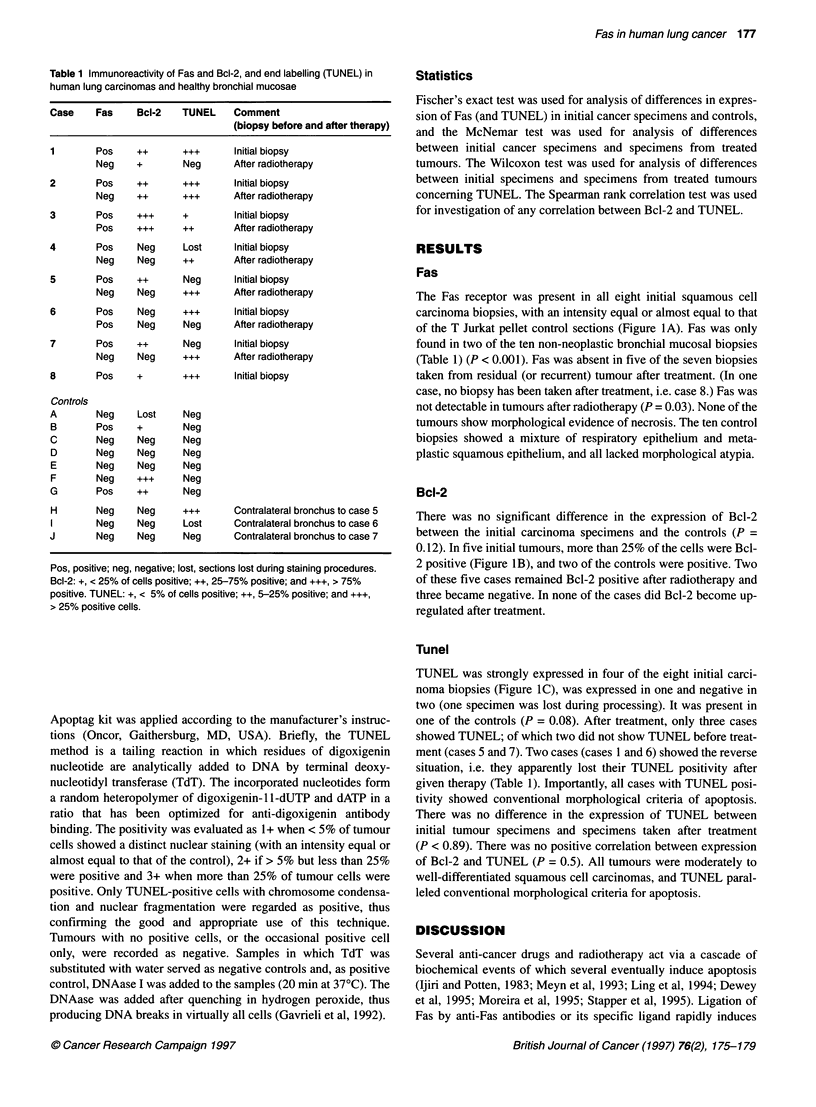

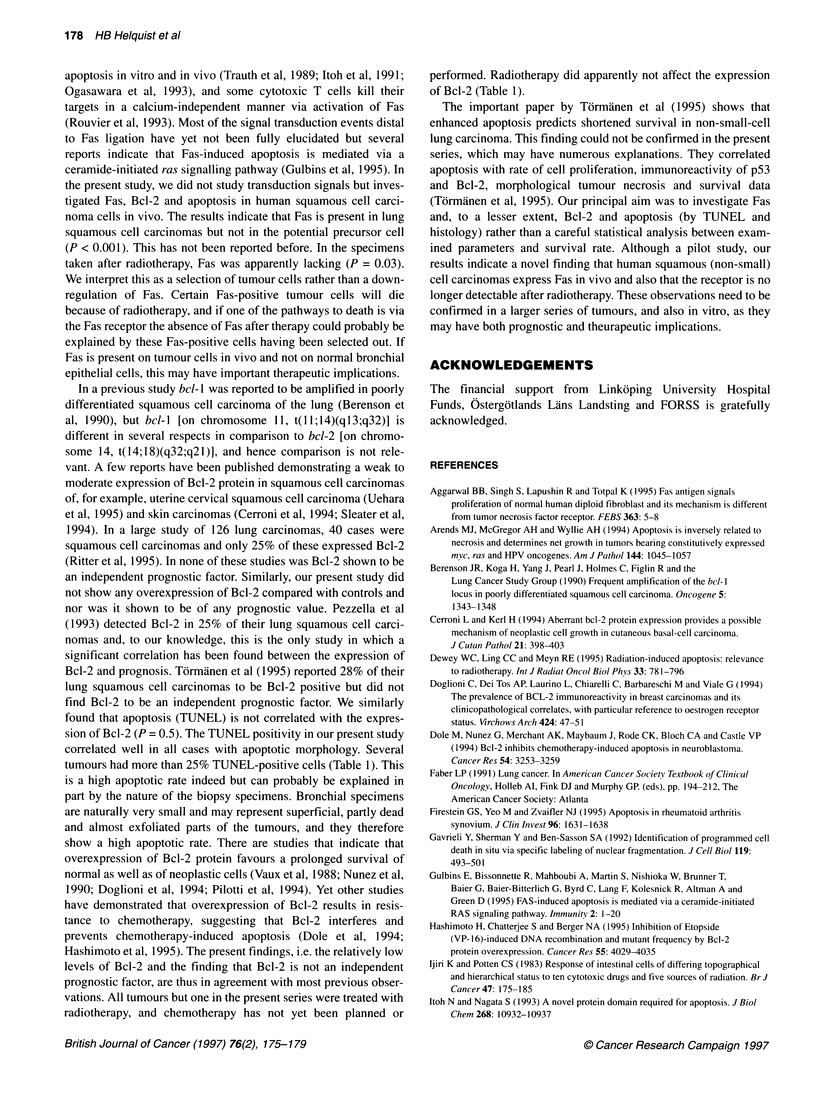

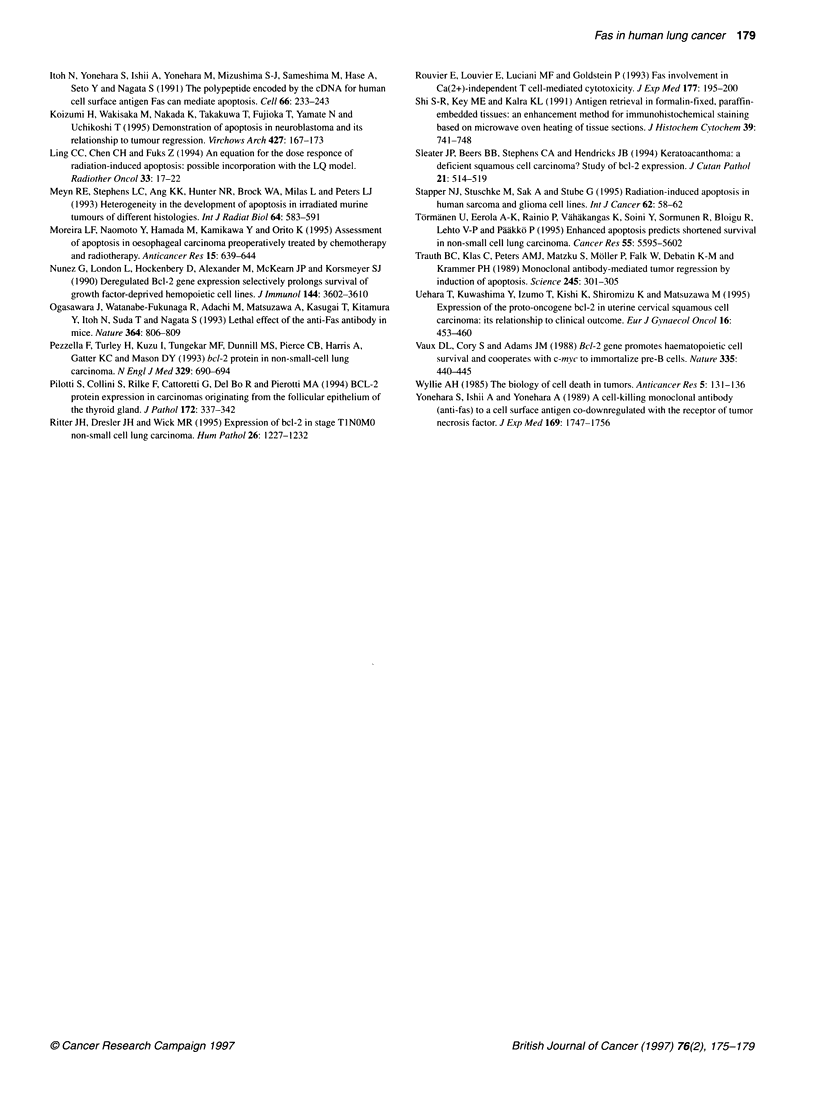


## References

[OCR_00424] Aggarwal B. B., Singh S., LaPushin R., Totpal K. (1995). Fas antigen signals proliferation of normal human diploid fibroblast and its mechanism is different from tumor necrosis factor receptor.. FEBS Lett.

[OCR_00429] Arends M. J., McGregor A. H., Wyllie A. H. (1994). Apoptosis is inversely related to necrosis and determines net growth in tumors bearing constitutively expressed myc, ras, and HPV oncogenes.. Am J Pathol.

[OCR_00433] Berenson J. R., Koga H., Yang J., Pearl J., Holmes E. C., Figlin R. (1990). Frequent amplification of the bcl-1 locus in poorly differentiated squamous cell carcinoma of the lung. The Lung Cancer Study Group.. Oncogene.

[OCR_00439] Cerroni L., Kerl H. (1994). Aberrant bcl-2 protein expression provides a possible mechanism of neoplastic cell growth in cutaneous basal-cell carcinoma.. J Cutan Pathol.

[OCR_00444] Dewey W. C., Ling C. C., Meyn R. E. (1995). Radiation-induced apoptosis: relevance to radiotherapy.. Int J Radiat Oncol Biol Phys.

[OCR_00448] Doglioni C., Dei Tos A. P., Laurino L., Chiarelli C., Barbareschi M., Viale G. (1994). The prevalence of BCL-2 immunoreactivity in breast carcinomas and its clinicopathological correlates, with particular reference to oestrogen receptor status.. Virchows Arch.

[OCR_00455] Dole M., Nuñez G., Merchant A. K., Maybaum J., Rode C. K., Bloch C. A., Castle V. P. (1994). Bcl-2 inhibits chemotherapy-induced apoptosis in neuroblastoma.. Cancer Res.

[OCR_00465] Firestein G. S., Yeo M., Zvaifler N. J. (1995). Apoptosis in rheumatoid arthritis synovium.. J Clin Invest.

[OCR_00469] Gavrieli Y., Sherman Y., Ben-Sasson S. A. (1992). Identification of programmed cell death in situ via specific labeling of nuclear DNA fragmentation.. J Cell Biol.

[OCR_00481] Hashimoto H., Chatterjee S., Berger N. A. (1995). Inhibition of etoposide (VP-16)-induced DNA recombination and mutant frequency by Bcl-2 protein overexpression.. Cancer Res.

[OCR_00486] Ijiri K., Potten C. S. (1983). Response of intestinal cells of differing topographical and hierarchical status to ten cytotoxic drugs and five sources of radiation.. Br J Cancer.

[OCR_00491] Itoh N., Nagata S. (1993). A novel protein domain required for apoptosis. Mutational analysis of human Fas antigen.. J Biol Chem.

[OCR_00499] Itoh N., Yonehara S., Ishii A., Yonehara M., Mizushima S., Sameshima M., Hase A., Seto Y., Nagata S. (1991). The polypeptide encoded by the cDNA for human cell surface antigen Fas can mediate apoptosis.. Cell.

[OCR_00474] Jameson S. C., Bevan M. J. (1995). T cell receptor antagonists and partial agonists.. Immunity.

[OCR_00504] Koizumi H., Wakisaka M., Nakada K., Takakuwa T., Fujioka T., Yamate N., Uchikoshi T. (1995). Demonstration of apoptosis in neuroblastoma and its relationship to tumour regression.. Virchows Arch.

[OCR_00509] Ling C. C., Chen C. H., Fuks Z. (1994). An equation for the dose response of radiation-induced apoptosis: possible incorporation with the LQ model.. Radiother Oncol.

[OCR_00514] Meyn R. E., Stephens L. C., Ang K. K., Hunter N. R., Brock W. A., Milas L., Peters L. J. (1993). Heterogeneity in the development of apoptosis in irradiated murine tumours of different histologies.. Int J Radiat Biol.

[OCR_00519] Moreira L. F., Naomoto Y., Hamada M., Kamikawa Y., Orita K. (1995). Assessment of apoptosis in oesophageal carcinoma preoperatively treated by chemotherapy and radiotherapy.. Anticancer Res.

[OCR_00524] Nuñez G., London L., Hockenbery D., Alexander M., McKearn J. P., Korsmeyer S. J. (1990). Deregulated Bcl-2 gene expression selectively prolongs survival of growth factor-deprived hemopoietic cell lines.. J Immunol.

[OCR_00529] Ogasawara J., Watanabe-Fukunaga R., Adachi M., Matsuzawa A., Kasugai T., Kitamura Y., Itoh N., Suda T., Nagata S. (1993). Lethal effect of the anti-Fas antibody in mice.. Nature.

[OCR_00534] Pezzella F., Turley H., Kuzu I., Tungekar M. F., Dunnill M. S., Pierce C. B., Harris A., Gatter K. C., Mason D. Y. (1993). bcl-2 protein in non-small-cell lung carcinoma.. N Engl J Med.

[OCR_00539] Pilotti S., Collini P., Rilke F., Cattoretti G., Del Bo R., Pierotti M. A. (1994). Bcl-2 protein expression in carcinomas originating from the follicular epithelium of the thyroid gland.. J Pathol.

[OCR_00544] Ritter J. H., Dresler C. M., Wick M. R. (1995). Expression of bcl-2 protein in stage T1N0M0 non-small cell lung carcinoma.. Hum Pathol.

[OCR_00548] Rouvier E., Luciani M. F., Golstein P. (1993). Fas involvement in Ca(2+)-independent T cell-mediated cytotoxicity.. J Exp Med.

[OCR_00552] Shi S. R., Key M. E., Kalra K. L. (1991). Antigen retrieval in formalin-fixed, paraffin-embedded tissues: an enhancement method for immunohistochemical staining based on microwave oven heating of tissue sections.. J Histochem Cytochem.

[OCR_00559] Sleater J. P., Beers B. B., Stephens C. A., Hendricks J. B. (1994). Keratoacanthoma: a deficient squamous cell carcinoma? Study of bcl-2 expression.. J Cutan Pathol.

[OCR_00564] Stapper N. J., Stuschke M., Sak A., Stüben G. (1995). Radiation-induced apoptosis in human sarcoma and glioma cell lines.. Int J Cancer.

[OCR_00573] Trauth B. C., Klas C., Peters A. M., Matzku S., Möller P., Falk W., Debatin K. M., Krammer P. H. (1989). Monoclonal antibody-mediated tumor regression by induction of apoptosis.. Science.

[OCR_00568] Törmänen U., Eerola A. K., Rainio P., Vähäkangas K., Soini Y., Sormunen R., Bloigu R., Lehto V. P., Päkkö P. (1995). Enhanced apoptosis predicts shortened survival in non-small cell lung carcinoma.. Cancer Res.

[OCR_00578] Uehara T., Kuwashima Y., Izumo T., Kishi K., Shiromizu K., Matsuzawa M. (1995). Expression of the proto-oncogene bcl-2 in uterine cervical squamous cell carcinoma: its relationship to clinical outcome.. Eur J Gynaecol Oncol.

[OCR_00584] Vaux D. L., Cory S., Adams J. M. (1988). Bcl-2 gene promotes haemopoietic cell survival and cooperates with c-myc to immortalize pre-B cells.. Nature.

[OCR_00589] Wyllie A. H. (1985). The biology of cell death in tumours.. Anticancer Res.

[OCR_00590] Yonehara S., Ishii A., Yonehara M. (1989). A cell-killing monoclonal antibody (anti-Fas) to a cell surface antigen co-downregulated with the receptor of tumor necrosis factor.. J Exp Med.

